# Using practical wisdom to facilitate ethical decision-making: a major empirical study of *phronesis* in the decision narratives of doctors

**DOI:** 10.1186/s12910-021-00581-y

**Published:** 2021-02-18

**Authors:** Mervyn Conroy, Aisha Y. Malik, Catherine Hale, Catherine Weir, Alan Brockie, Chris Turner

**Affiliations:** 1 Health Services Management Centre, University of Birmingham, Birmingham, UK; 2 Warwick Medical School, University of Warwick, Coventry, UK; 3 Emergency Medicine, University Hospitals Coventry and Warwickshire, Coventry, UK; 4 Department for Continuing Education, University of Oxford, Oxford, UK

**Keywords:** *Phronesis*, Practical wisdom, Practice virtue ethics, Decision-making, Clinical leadership, Neo-Aristotelean

## Abstract

**Background:**

Medical ethics has recently seen a drive away from multiple prescriptive approaches, where physicians are inundated with guidelines and principles, towards alternative, less deontological perspectives. This represents a clear call for theory building that does not produce more guidelines. *Phronesis* (practical wisdom) offers an alternative approach for ethical decision-making based on an application of accumulated wisdom gained through previous practice dilemmas and decisions experienced by practitioners. *Phronesis,* as an ‘executive virtue’, offers a way to navigate the practice virtues for any given case to reach a final decision on the way forward. However, very limited empirical data exist to support the theory of *phronesis*-based medical decision-making, and what does exist tends to focus on individual practitioners rather than practice-based communities of physicians.

**Methods:**

The primary research question was: What does it mean to medical practitioners to make ethically wise decisions for patients and their communities? A three-year ethnographic study explored the practical wisdom of doctors (n = 131) and used their narratives to develop theoretical understanding of the concepts of ethical decision-making. Data collection included narrative interviews and observations with hospital doctors and General Practitioners at all stages in career progression. The analysis draws on neo-Aristotelian, MacIntyrean concepts of practice- based virtue ethics and was supported by an arts-based film production process.

**Results:**

We found that individually doctors conveyed many different practice virtues and those were consolidated into fifteen virtue continua that convey the participants’ ‘collective practical wisdom’, including the *phronesis* virtue. This study advances the existing theory and practice on *phronesis* as a decision-making approach due to the availability of these continua.

**Conclusion:**

Given the arguments that doctors feel professionally and personally vulnerable in the context of ethical decision-making, the continua in the form of a video series and app based moral debating resource can support before, during and after decision-making reflection. The potential implications are that these theoretical findings can be used by educators and practitioners as a non-prescriptive alternative to improve ethical decision-making, thereby addressing the call in the literature, and benefit patients and their communities, as well.

**Supplementary Information:**

The online version contains supplementary material available at 10.1186/s12910-021-00581-y.

## Background

The health and well-being of people is dependent on medical and public health decision-making. In recent years, there have been calls for using *phronesis* (practical wisdom) for medical-related ethical decision-making as a complement to evidence-based practice [1]. This is because the latter does not consider the particularities of any given case and context.

Although Gallagher et al. [2] argue that doctors are motivated primarily by deontological (guideline or rule-based) approaches to decision-making in the context of medical practice, the quantity of such guidelines means that this prescriptive approach has become unmanageable. One estimate suggests that there may be more than 7000 deontological guidelines for clinicians to follow, with many being added every year [3]. Practitioners note that the growing use of ever-closer codification of medical practice is not able to take into account the complexity of caring for patients with multiple comorbidities and within difficult contexts [4]. The abstractness of principle-based approaches raises concerns about their utility in decision making in complex clinical context [5, 6]. Further, Torjuul et al. [7] suggest that meeting the expectations of patients, colleagues and society makes doctors professionally and personally vulnerable. For instance, although doctors’ main concern are their patients, in an environment of resource and regulatory constraints, meeting patients’ expectations and distributing health care is challenging [7]. We argue that the theory presented in this paper offers a partial ‘antidote’ that makes the process of ethical decision-making easier, potentially reducing feelings of vulnerability and can build physician resilience.

Our research responds to the call for overcoming the limitations of the dominant deontology-based approach and meets the need for a new approach with a conceptual framework used as a moral debating resource for cultivating *phronesis* (practical wisdom) in the process of making ethical decisions.

*Phronesis* is a conceptual approach to ethical decision-making grounded in an accumulated wisdom gained through previous practice dilemmas and decisions. In effect, *phronesis* is an “executive virtue” that keeps stakeholders central to the decision-making process, allowing ethical choices to be executed in practice [8]. In their account of ethical decision-making, Jonsen et al. [9] report that the fundamental difficulty in all clinical settings is uncertainty, noting that the biological side of the uncertainty problem is severe because it is often very hard to be sure of a diagnosis or be certain that a specific treatment will work. Additional layers of uncertainty further compound this situation. For instance: What do patients prefer? Do they value length or quality of life? Do they prefer treatment (or no treatment) in line with religious or moral beliefs? Moreover, understanding the views of interested parties is essential: Does the patient’s family have wishes? Does the hospital or health system have certain priorities of what can and cannot be treated due to financial constraints? Regardless of such preferences, what *is* the right or wrong thing to do? What if patients, relatives and hospitals prefer courses of treatment (such as antibiotics) that are not in the collective interest of the community to give?

When different moral and social problems begin to interact with each another, medical decision-makers may not be certain about what is best for patients and society, and how best to achieve optimal outcomes for both, simultaneously. Decision-makers must choose that what is in the best interest of patients and society is also in line with good clinical practice and has a good chance of working in practice.

Toulmin [10] and MacIntyre [11] align with each other in arguing for a recovery of the virtue of practical judgement to overcome disagreements and conflicts in the form of Aristotle’s *phronesis* [12]. In Aristotle’s work, *phronesis* is the intellectual virtue that helps turn one’s moral instincts into practical moral action [13] by providing the practical know-how needed to turn virtue into successful action and enables *phronimos* to weigh up the importance of different virtues and competing goals in a given moral situation [14]. While moral virtues enable us to achieve the end, *phronesis*, makes us adopt the right means to that end [13:161]. Both moral virtues and *phronesis* work in tandem. In the absence of the former, *phronesis* degenerates into a “certain cunning capacity for linking means to any end rather than to those ends that are genuine goods for man” [11]:154]. Whereas, in the absence of *phronesis*, we may be lost in the moral maze.

Building on Jonsen and Toulmin [15] a number of authors [16–20] have developed theoretical accounts of judgement in medicine based on Aristotle’s account of *phronesis* being the kind of practical wisdom that is needed to promote the good in morally difficult situations.

Pellegrino and Thomasma [16] provide the most forceful defence of the virtue-theoretic approach to medical ethics. They write that rule, or principle-based approaches to medical ethics are “too abstract” and “too formularized and far removed from the concrete human particulars of moral choice” [16]:19]. Alternatively, *phronesis* being medicine’s “indispensable virtue” plays a crucial role in providing an essential connection between seeing or understanding what is right or good and knowing how to do good [16]:84].

Because different virtues can pull doctors in different directions (e.g. compassion drives her to give an optimistic assessment to a patient and honesty drives her to tell the truth), *phronesis* helps put virtues in the “proper order of priority and to make the right and good decision in the most difficult situation” [21]:382]. In the ‘people professions’ the culture of mere compliance to rules and guidelines [14, 22] tends to oversimplify the complex clinical situation, making patients single-pathology entities rather than the multifaceted (medically and socially) humans they are, who require a holistic approach. Focussing on the patient as a *person* is imperative; science alone is not enough to understand the complexity of the case [21, 23, 24]. A recurrent theme of practice virtues being argued for as important is made in Carr et al. [25] and Kristjansson [14], and further supported by Jonsen and Toulmin [15], Pellegrino and Thomasma [16], Pellegrino[21], Montgomery [18], Toon[19] and Kaldjian [17].

Although there has been a drive to theorise about the importance of empirically-informed ethics [26–28], attempts to study clinical-judgement as *phronesis* using empirical data are rare. Notable exceptions are Jordens and Little [29], Little et al*.* [30], Jones et al. [31] and Conroy et al. [32]. However, all of these studies have been limited by small sample sizes. A clear gap existed for a large empirical study to inform the development of a virtue-based *phronetic* approach to medical and public health decision making.

We use *phronesis* as a theoretical frame to analyse the narratives and the observations on medical decisions that our participants conveyed both as wise and not so wise for patients and their community. Being within the neo-Aristotelean practice virtue ethics theoretical framework ([11], the concept of *phronesis* includes the telos of well-being for all in society.

We acknowledge other alternatives to deontological reasoning such as casuistry, narrative ethics and discourse ethics [e.g. 33]. The choice to focus on a practice virtue ethics approach came from a combination of the gap and emphasis in the literature, as outlined above, and directly from feedback from policy makers, academics and practitioners at the first workshop for this study [8]

The first step in practice virtue ethics is to understand the virtues that are considered by communities of practitioners, in this case the medical practice, to be important for their practice. Real cases communicated through stories or narratives, according to Montgomery [18], offer the best approach to eliciting practice virtues. The ontology of narrative conveying and transmitting practice virtues is supported by MacIntyre [11]. Therefore, to fill the gap in understanding, outlined above, we started our research with the following questions:

1. What does it mean to the medical community to make ethically wise decisions?
2. What virtues do the medical community convey as important for decision-making?
3. What approach do medical practitioners use to navigate the virtues they consider important when making decisions?

These questions formed the basis of a three-year study (2015–2018), ‘*Phronesis* and the Medical Community’ (PMC), which used a narrative and arts-based methodology to understand what it means to a community of UK medical practitioners to make ethically wise decisions for patients and their communities. Narratives from all medical career stages were collected. Following Kaldjian [17] and MacIntyre [11], we critically examined the practice narratives with particular attention to the virtues conveyed.

This paper describes the methodology, summarises our findings, discusses their contribution to ethically wise decision-making and suggests implications for medical education, policy and research.

## Methods

Our research methods are based upon three main sources. From the humanities, we draw on the practice virtues philosophy of MacIntyre [11] and his argument that practitioner narratives convey the virtues of the practice of interest: narratives “humanize” situations and convey “what matters to us” [34, 35]. From social sciences, we use Flyvbjerg et al.’s [36] *phronesis*-based ethnography to contextualize the collected stories. From the arts, we draw on a participatory video production approach [37], where the research team and some of the participants are involved in iteratively producing a film series and app that convey the collective set of virtues derived from the narratives [38].

Our data sources were in-depth, semi-structured interviews and direct observation of medical decision-making at multi-disciplinary team meetings (MDT). This approach allowed us to collect narratives and consider contexts, which indicated what participants considered to be ethically wise decisions and those which they considered to be unwise for their patients and communities. Our methods and data sources are appropriate to answering our three research questions and build on those used in other studies with an interest of understanding practice virtues [e.g. 39]. We draw on the language used by the participants when they refer to their practice and then changed that to a practice virtue. For example, practitioners may refer to ‘negotiates’ and that is interpreted as an action referring to practitioners negotiating with patients and the equivalent practice virtue would be ‘negotiation’.

### Data collection

Three communities of doctors were interviewed from mid-2015 to early 2017. Interviews took place during three time periods:

1. Beginning of formal medical study (2nd year) and end of formal medical study (5th/final year)
2. On placement at the end of formal study, Foundation year 1(FY1) and Foundation Year 2 (FY2) and then in GP or Consultant traineeship.
3. Established GPs and medical consultants with 5 years or more experience.

131 doctors were interviewed. Table 1 shows the number of participants per cohort.

**Table 1:** Number of participants per cohort

2nd year	27
5th year	25
FY 1	28
FY 2	20
Experienced	31
Total	131

Ethics approval was obtained from the Science, Technology, Engineering and Mathematics Ethical Review Committee, University of Birmingham (reference ERN_15-0172) and the Health Research Authority REC (reference: 18/HRA/0203) for observations.

Eligible doctors and medical students were identified with the help of the academic and administration co-ordinators at the three participating medical schools (of University of Birmingham, University of Warwick and University of Nottingham)  and their local NHS Trust hospitals. Thereafter, using a snowball sampling technique, more doctors were approached and invited to participate via emails. All communication between interested potential participants and researchers was treated as confidential. Emails did not reveal who else was contacted. All medical students, FY1, FY2 doctors and experienced doctors who were interviewed were asked if they would consent to observations. Observations were carried out from August 2017 to November 2017.

Of the 48 FY (1 and 2) cohort 13 were interviewed twice, first when they were in FY 1 and re-interviewed when they moved to FY2, to understand to what extent their *phronesis* develops as they move from FY1 to FY2. Non-participatory observations were conducted when four experienced practitioners, were working with multidisciplinary teams or peer groups to make decisions for their patients.

Participants were sent participant information sheets. Those who consented were invited for an interview. Semi-structured interviews (SSI) were conducted that developed from story gathering methods [39]. The interviewer started by explaining that we were interested in exploring: (i) the participant’s experience of involvement in making ethical/wise decisions; (ii) whether their own or those of others they work with; (iii) whether they perceived them to be good/wise or not so good/unwise decisions. Although an *aide memoire* was prepared, to be used as prompts only, participants started their accounts wherever and however they wanted. If they did not respond to this open start, the interviewer might prompt with ‘It seems difficult for you to start on this subject, would you like to start by talking about the difficulty?’ They were then asked about the instances or stories that they subsequently alluded to and so, the interview built on participants’ responses. [8]

Most interviews were conducted face to face at the medical schools for the medical students, for the practicing doctors they were conducted at the hospitals where they worked, after they had completed their clinical duties, while a few interviews were conducted via telephone. The interviews, which lasted between 40 and 60 min, were audio–recorded and transcribed verbatim. Four FY participants and two experienced doctors from across the three sites provided a diary focusing on experiences of meaningfulness and puzzlement that they indicated represented practical wisdom (wise or unwise decisions). The narratives were used to generate discussion and along with the observations provided the basis for storyboards and scripts that led to the production of a video series. Prior to video production the scripts were fed back to some of the doctors (two consultants, two GPs and a FY doctor) who attended a steering group meeting to ensure they were a fair and balanced representation of the kind of stories that circulate in their practice environment. Subsequently the alpha version of the videos produced were reviewed by nineteen medical practitioners (Consultants and GPs) medical ethics’ tutors and CPD providers at a workshop, held in March 2018. The feedback from workshop participants was collated and sent to the film production team for an update to a beta version of video series [38].

### Data analysis

Interview data were analysed thematically by reading and rereading the transcripts to make the transition from literal meaning to practice virtue themes. Data were organized and coded using NVivo 11 Plus. Coding and analysis were conducted simultaneously. The research team met regularly, initially to discuss coding strategies and categories and then to check (inter-coder reliability) and to consolidate virtue themes.

The nature of the practice virtues including *phronesis* was examined through two theoretical lenses: MacIntyre’s [11] practice based virtue ethics and Kaldjian’s [40] medical *phronesis*. Findings from the latter are presented elsewhere [41].

After four iterations, a set of 15 virtues were agreed. These 15 virtues were put into virtue continua (Table 2). This consolidated set of virtues, along with the narratives and observations, were used as the basis of the video series.

**Table 2:** Virtue Continua

Virtue (V.)	POLE1(excess)	MEAN	POLE2 (deficiency)
1	Doctor decides	Negotiation	Patient decides
2	ALL get treatment	Justice/Fairness	Select few get treated
3	Overly trusted	Trustworthiness/(including maintaining confidentiality)	No trust
4	Constant litigation worry	Lawfulness	Ignore legal constraints
5	Constantly seeks Guidance from peers and/or professional bodies	Collaboration (Making Collaborative Decisions/Seek guidance)	Self –guided/Does not consult
6	Use own values and beliefs	Cultural competence	Go with patient’s values and beliefs only
7	Too involved (Over emotional)	Emotional Intelligence (including Interpersonal Communication)	Distant/Aloof
8	Treat at all cost	Awareness of limits to treatments	Limited consideration of treatment options
9	Constant mentoring/overly directive	Mentorship (and being an approachable mentor)	No interest in mentorship
10	Trying to cater to all aspects – Arts/humanities science, spiritual and physical	Balanced approach	Just one approach—e.g. science/clinical only
11	Over-analytical (Navel gazing)	Reflexive	Never reflect
12	Foolhardy risk taker	Courage (to speak out and act)	Cowardice (Avoids conflict)
13	Thinking they are bullet proof	Resilience	Avoidance of any stress
14	Obsessed with finances/resources	Resource awareness	No consideration of resources
15	Seen it all/know it all/can deal with anything	Phronesis	Applies purely theory or just follows guidelines

Use of combined narrative and film production methodology enabled MacIntyre’s [11] practice virtue ethics to emerge from our data as the ‘collective practical wisdom’: a non-prescriptive moral debating resource, that reflects what it means to participants to make ethical decisions that contribute to the good for patients and their communities. The close fit between our data and practice virtue ethics concepts in video and app format, including *phronesis,* means they offer a new and accessible resource for doctors at all stages in their professional careers. Practitioners may find the resource aids ethical reflection about their decision-making, as a guide to ethical action, and as a foundation for resolving discrepancies between virtues.

## Results

Participants conveyed many different practice virtues but no one participant conveyed all 15 virtues. The more experienced participants conveyed more of the virtues than the less experienced participants. This finding connects to the neo-Aristotelian framework of practice virtues constructed by MacIntyre [11] and advocated by Carr [42]. MacIntyre suggests determining the practice virtues across a peer group of practitioners rather than individual moral characters. This fits with the notion of diversity in practitioner contributions bringing a more robust set of virtues to the practice [25, 42]. The consolidated 15 virtues we present here represent a “starter-set” of virtues for medical practice that are open for debate and challenge from others. As a non-prescriptive debating resource, this combined *phronesis* offers a powerful way for those in medical education and practice to debate forms of decision-making to serve the best interest of patients and their communities. The arts-based element of the analysis was used to produce a seven-part video series, which is an enacted form of our participants’ ‘collective practical wisdom’. The videos offer a highly accessible form of moral debating resource for reflection before, during and after medical decision-making (for an example see episode 3 at: https://www.youtube.com/watch?v=Azkxeddnlpg).

### Virtue continua

We present our findings as a “virtue continua” (Table 2) before presenting them in text form. The virtue continua show the virtues conveyed by our interviewees in their narratives. Each virtue extends from pole-to-pole via a mean.

Here we present four examples from Table 2, above, to demonstrate how the data supported the analysis. Additional file 1 shows all the 15 virtues gleaned from the participants’ narratives and Additional file 2 the virtues from observation data.

#### Negotiation (V.1)

Many participants produced narratives about negotiating with patients and others when making decisions about treatment. Some conveyed the doctor’s role as providing suitable and relevant information to enable patients/carers to come to a decision. However, other narratives reflected that providing expert advice and guidance in the light of clinical facts was important to patients while also taking patients’ views into account. This enabled informed choice in partnership with the patient:

I guess that would be my approach, just to seek out as many facts as I possibly could on the one hand, and for more… difficult decisions, just talking to the patient and trying to get to know them a bit better and their kind of particular outlook ….. and then possibly based on that, kind of guide them to a decision that I think might suit them better. (B102).

Experienced doctors spoke about the importance of dialogue and how exchanging information resolves conflicts and enables patients to make an informed choice:

[A] a constructive conversation both ways. I’ve got something to say but let’s not jump to a decision now, because that would be wrong. (BX02).

However, decisiveness was respected both by doctors-in-training and by some patients they encountered. It was reported that some patients implicitly sought paternalistic guidance, as they may find decision-making burdensome, relying on the doctor’s expertise and knowledge to guide them:

Sometimes people do respond well actually to someone taking control of the situation, even if it’s in a way that you would think would surely be completely inappropriate, but [the patients] respond well to it. (B112).

A FY doctor reflected (in their diary) on the distress caused knowing fully well that if a surgical procedure is not conducted it would prove fatal for the patient but agreed to the request of  the patient:

I felt very sorry for the patient, and a little disturbed at the idea that this was almost certainly going to kill her…… I therefore found it difficult to reconcile my wish to respect her autonomy and her decision making, and the horrific consequences of her choice. (W 108).

Sometimes, some participants said that persuading patients in their best interest is necessary because:

[A] patient doesn’t understand the severity of the decision they’re making, and perhaps only when they’ve seen people who don’t have the procedure done or don’t have an operation might they learn… the actual nature of the decision they’re making, because we see it, whereas they don’t. (WX02).

Sometimes doctors, led by patient autonomy, assume the role of information providers, enabling patients’ decisions to be implemented:

But, for me, a good decision is one where the patient is the one who essentially makes the decision, or puts forward their wishes, and we then, as the clinicians, allow that decision to come to fruition. (B107).

#### Collaboration (Being Collaborative/seeking guidance) (V.5)

Many participants narrated stories about the present-day clinical paradigm being where professionally isolated decision-making is often neither advisable nor possible. Seeking to involve all those entrusted with a particular patient’s care allowed holistic, tailored decisions. Counsel from multiple parties and professional guidelines was reported to be valuable. This was corroborated by our observations of different MDT meetings. When making decisions for complex cases, team members frequently found that the progressive decisions reached and displayed on the whiteboards were useful, as “they help prioritise and review decisions.” (Obs.1).

Guidelines, though useful, require contextual awareness that can be provided by those who know the patient well, such as:

[T]he nursing staff who cared for the patient throughout, I relied on hugely …and even the night sister … just made it more logical, and decision-making more logical. I do rely on my consultants for the ultimate decision quite a lot of the time. (BX01).

In observation 2, the roles of the occupational therapist (OT), physiotherapist (PT) and speech therapist were seen to be central to certain patients’ treatment because they had the most up-to-date information. The Registrars and Consultant relied on the OT and PT to provide almost the whole information summary. These collaborative discussions become critically important when making “deprivation of liberty” decisions. This observation made it clear that:

The lead consultant would ask questions and appeared to be kind of taking it all in, cross-referencing information he got with his records on his computer. More often than not, he would defer to the decisions of the PT and OT…..The nurse had a lot of say as well about how patients were progressing towards their goals. (Obs. 2).

This approach was not universal, though. At another MDT observation (Obs. 3), the discussions were mostly contained amongst the doctors, with barely any input from other staff.

Most medical students were of the view that it is far better for “not-so-experienced doctors” to defer to people with more experience:

[Y]ou know, bigger decisions, you’re not going to want to take that onto yourself, you’re going to defer to people that have got the experience. (W203)

Several newly trained doctors conveyed that they appreciated the opportunities to seek guidance from, and feel reassured by, more experienced doctors. This was observed (Obs. 3) in an Emergency Department environment, when the junior doctor requested the  Consultant to discuss “an older patient with complex health and social problems”, because:

…. they’ve probably made that [decision] before and they can tell you with experience the outcome and why. And they might come up with ideas as to why your idea might not be the best for that patient. (W101).

Experienced doctors also conveyed that they seek guidance in challenging cases as it helps them clarify the situation and make better decisions:

…sometimes that consensus is really useful because you’re basically going through the arguments … and again clarifying some of the aspects of it, I think. (BX11)

Some participants referred to guidelines being interpreted contextually. This could result in referring to more experienced doctors to gain insights into wider interpretations of the guidelines:

…so we’ve got an SHO ….[with] very good book knowledge, he’s very academic, [and] knows the guidelines for everything off by heart but he doesn’t really have a grasp of the fact that not every patient can be treated as per guidelines. And we’ve been trying to explain to him that a guideline is just that, it’s a guideline, it’s not rigid; it’s meant to guide your practice….. he’s had real issues with not calling for senior support because he feels that he’s got a guideline to follow and that he follows it.. (WX05).

#### Cultural competence (V.6)

Some stories conveyed that respecting patients’ values and beliefs is important. Many of the participants said that they consult their colleagues to understand cultural issues. However, some participants narrated experiences where the doctor chose to follow their own beliefs and values, rather than the patients’. One doctor experienced a situation where a doctor refused an intervention that challenged their personal beliefs leading to treatment delay. They said:

[I]t is important to park your own values. You should not allow those values to affect the decision. (BX04).

Similarly, reflecting in their diary a FY doctor wrote:

However, one point I am completely certain of is the importance of professionalism. I feel one should never impose their views on to a patient (W103).

A 5th Year medical student told of a consultation in a sexual health clinic, where the doctor seemed judgemental towards a patient:

[H]e said something like, ‘Are you gay or straight?’ or something. Just, like, which is incorrectly phrased? There’s far more, like, tactful ways to do it. But he, kind of, shouted at them, so, ‘Are you gay?’ kind of thing. (B501).

Cross-cultural sensitivity was stated to be important in building trust. Rehabilitation, for example, is conveyed as following a “white Anglo-Saxon” model. An experienced doctor explained:

I think there’s – generally there’s mistrust of the NHS that we pull out too soon, and we don’t do everything that’s possible,…………. don’t do everything we should be doing.. but that’s compounded by a cultural view of life I think….. there is[at times] a cultural clash, so there is mistrust that can be on both sides. The only way to get around that is to recognise that there is a difference in view and maintain open dialogue. (BX05).

#### Emotional Intelligence (including Interpersonal communication) (V.7)

Good interpersonal communication was conveyed as commendable by our participants, because:

[Y]ou can be the greatest doctor in the world but if you can’t communicate, nobody will do what you say, will they? (BX103).

However, some conveyed that having the clinical knowledge regarding the disease is also essential:

[Y]ou can be a very compassionate person, but a useless doctor if you don’t know what you’re doing. (W207),

Some participants narrated experiences where an apparent lack of interpersonal communication skills was displayed by a doctor:

…the clinician who saw her [the patient] wasn’t very communicative and reassuring in his approach to the patient… [the patient] was having a miscarriage, [the clinician] left it at that; left the room, and I was standing there with a very distraught couple… I told the clinician and he said, ‘Oh they’ll probably figure it out some way along the line’. And wasn’t very keen on going back and telling the patient – reassuring them**.** (W502).

Others conveyed how empathic communication made patients amenable to discussion:

I think you have to, I suppose, temper your objective, rational facts for your decision-making process in a way that comes across as empathetic and sympathetic and looking at a bigger picture beyond the current situation; and also to help parents to think about things from their baby's view. (BX12).

#### Phronesis (V.15)

The development of practical wisdom was conveyed by most interviewees as sequentially experiential. One medical student termed it *“learned experience”* (N203), while a FY doctor spoke of it as a *“mix of nurture and nature”* (B104, Follow-up). For one experienced doctor:

…some people are inherently wiser, they are really wise people…now, whether that wisdom is inherent or … is simply because that person has walked past that journey ahead of me. (NX05).

Experience can, however, lead to an assumption about personal knowledge. Another FY doctor recounted how the  Consultant seemed to show a lack of compassion and so:

“… experience makes you better at making clinical decisions… but not necessarily in terms of ethical decisions… a lot of people get stuck in their ways. (B504)

Assuming that they “know it all” and following a textbook approach can, according to another participant, cause a doctor to be caught out:

You can’t make a decision based on what the textbooks say… because if the textbooks say it, you can only say that that’s right 99% of the time. There will always be the one case that will catch you out if you treat everybody the same… there’s things that are really rare, but they still happen. (WX02).

An experienced doctor reported another risk that arises with experience and seniority as being “*arrogant or foolhardy.” (BX04)*. Similarly, another experienced doctor reported on a senior consultant who regularly over-ruled based on experience rather than book knowledge*:*

Because evidence-based medicine tells you something else, but the experience of this doctor was something different, so there is, kind of, a clash between the two, rather than both going forward in a symbiotic relationship…. Which is why I'm wary of saying that wisdom is the most important thing. (NX04).

*Phronesis* was variously described as the collation of holistic information, both clinical and social, from different sources, as well as being able to weigh that up against protocols, guidelines, various situations encountered in the past and then getting other “opinions, other approval, putting the situation to a new pair of eyes, and saying okay this is what I have got here.” (B106).

But for some medical students, *phronesis* seemed to be narrowly defined as diagnostic skills (i.e. *techne*) as opposed to the broader process of a holistic decision-making (as described by FY and experienced doctors):

You know you learn by example, by following what someone else is doing… the art and the science of medicine… you need the clinical knowledge and then you need the experience to know how to apply it and when to apply it. (W207).

Another medical student spoke of consultants with a “repertoire of patterns” (W209). However, *e*xperience and “time served” were not enough to guarantee wise decision-making, and certain other virtues were seen as key to phronetic decisions. For instance, being reflective, “open to insight” (WX04) and being consultative: “ always questioning what the right thing to do is.” (B110). There were those who considered it as intuition, “a sixth sense” (NX02).

*Phronetic* decisions were often seen as the avoidance of the rigid application of rules and guidelines, what was termed by one experienced doctor as the “protocolisation of medicine” (WX03). In medicine “it is this difficulty of managing an illness rather than treatment of an illness which is the more difficult bit and there are never going to be mathematically accurate answers.” (WX06).

*Phronetic* decisions were often conveyed as practical and experiential:

… where wisdom comes from, it’s a lot of thinking back to your past experiences and what you did badly, what you did well and trying to apply that… You’ve just got to think about in an ideal world what you want to do, and then think how you could possibly get as close to that with what you’ve got. (B110 FP).

## Discussion

The virtue continua in Table 2 show fifteen virtues, including *phronesis*, and the spectrum of actions for each, from deficiency to excess via a mean. This table represents the consolidated ‘collective practical wisdom’ conveyed in the decision narratives of the study participants. These fifteen virtues were conveyed in small sets or individually in the narratives collected from medical practitioners. Thus, they combine diversity of thinking and experience across the medical community interviewed and observed. These practice virtues represent a collective of what is required to come to wise decisions that are best for patients and their communities.

However, we are not claiming that they represent a final list of virtues that all practitioners should be following. Instead, they are formulated as a response to MacIntyre’s [43] identification of a vital but missing component in professional education (including medics and other health professionals) of moral debating resources. The shared 15 virtue continua provide and act as exactly that, a stimulus for reflection and moral debate. We therefore argue that it is now possible to cultivate *phronesis* through that reflection and debate, to support the process of arriving at decisions that are right for the range of cases and contexts that practitioners are faced with.

These findings provide empirical evidence to support the theory that good practice emerges from agreeing the virtues across a group of people who conduct that practice [20, 44, 45]. Pellegrino and Thomasma write about the virtues of compassion, prudence, justice, trust, fortitude, temperance, integrity, respect and benevolence [16]. In her encounters with empathetic doctors, Kristiansen, narrates how the humanity of doctors helped make sense of the suffering and loss she experienced, long after the clinical encounter had ended [46]. The flexibility of this new virtue continua allows practitioners with a preference for these virtues to be included or merged with one of the fifteen presented in Table 2. Interestingly this was reported by some of the research participants as part of ‘collaboration’ virtue (V.5 in Table 2), in that collaboration needs to exist not just with other practitioners but with any other guidance from professional bodies or other sources such as academic literature on the topic. This may lead to apparent disjunctions between virtues put forward in the fifteen here and those from other sources. However, since these new continua are not prescriptive, i.e. ‘this is how it *should* be done’, but instead a stimulus for moral debate, this would mean that the resource is fulfilling the role of providing a moral debating resource. It is then up to the community of practitioners to decide what virtues and continua are of relevance to the case, or cases, at hand. Thus, this is not a case of empirical trumps theoretical, or any other guideline for that matter, but simply a way of cultivating practical wisdom within and across practices through the moral debate that ensues. The flexibility in the continua allows for the two to be integrated, which is the role they are designed to fulfil to ensure that the diversity in the community is fully synthesised.

This also applies to community-based moral reasoning which may on occasion generate tension with individual reasoning. Participants consider that ethically wise decisions are guided by their medical knowledge (*techne*) and virtues, including the ability to understand patients’ values [47]. Participants conveyed that negotiating treatment plans was important; although some reported relational interdependence, grounded in relational ethics, played an important role in making practically wise decisions [24, 48]. Participants also suggested it is helpful to collaborate with those who know the context and can advise whether the decisions made are applicable in the real-world [1]. They indicated that seeking guidance on making decisions, especially in complex situations, from colleagues either senior or peers who have experiential knowledge, provides reassurance. This notion is corroborated by the nursing profession [49, 50]. Resource constraints (time and finances) were reported as affecting communication and decisions made, and “provide the conditions in which unsafe acts occur” [51]. Worries about litigation (“covering myself”) also had a tangible effect on decisions [52]. Resolving that type of tension through moral debate could be facilitated by the resources.

### Implications for practice

The findings answer a call in the literature for a moral debating resource to support practice virtue ethics/*phronesis*-based approach for ethical decision-making in medical practice. This is despite the detractors of such an approach who argue that this is an unreachable ideal and *phronesis* is conceptualised in different forms [e.g. 14]. The form of conceptualisation used is the practice virtue ethics version as advocated by MacIntyre [11] and supported by others [e.g. 17–19] in medically contextualised studies. Furthermore, given the argument that the role of doctors is changing from being the sole guardians of medical knowledge [53, 54] to being facilitators of practically wise decisions, weight is given to this approach being conceptually and practically relevant right now.

We argue these fifteen virtues represent an intra-medical practice collective for the doctors interviewed and observed (n = 131). One practitioner possessing all these virtues in their character is an unrealistic ideal, or as Curzer states: “one person can have some but not all the virtues” [55]:70]. Therefore, rather than them being a set of individual character virtues in the original Aristotelian tradition, we argue that the table provides their collective ethos and so aligns with the neo-Aristotelian concept of practice virtue ethics.

In this collective form and when conveyed in the highly accessible video series and online App the 15 virtues provide a form of practical wisdom that can be used in both professional education and practice for moral debate related to ethical decision-making [56]. This supports the notion that such an approach leads towards professional fulfilment and practice excellence [11]. The flexibility of the continua allows *phronesis* to be cultivated within practice and across related practices in the wider community; which according to MacIntyre [11] is also part of schema to establish virtuous practices and a *telos* (purpose) of well-being for all in the community.

A virtuous act “hits the target” by deriving an understanding of the situation and acknowledging all its pertinent features [22]:11] and in so doing requires moral judgement to discern how to act wisely. Our findings mean practice virtue ethics and *phronesis* can be used to complement other ethical approaches and clinical knowledge, leading to treatment plans/decisions that consider the particularities in each case. This is integral to reaching a diagnosis and proposing a plan of care that gives primacy to the best interest of the patient, and the community. Our findings emphasize that even when virtues are recognised for that particular practice (e.g. negotiation, reflection, cultural competence, collaboration, recognising limits to treatment etc.), knowing where to act on the continuum requires discernment that is provided by the intellectual virtue of *phronesis* [11].

So that moral reasoning and relevance work simultaneously [57], this framework offers practitioners a helpful stimulus to achieve that. On this view, we agree with others about introducing practical wisdom during the “formative development of medical students’ ethical reasoning” [58, 59]:241] which also enriches teaching methods for ethics [60].

Cribb argues that to deliver independent rigorous (moral) thinking, relevant to the situation at hand, without sacrificing rigour for relevance, is a challenge for translational ethics [57]. By consolidating the 15 virtue continua as ‘Collective Practical Wisdom’ and conveying them in an enacted seven-part video series we created a contemporary moral debating resource that responds well to the challenge of translational ethics identified by Cribb [57] and is applicable in varied contexts. As stated earlier, rather than a deontological prescription as the definitive, ‘this is how it *should* be done’, the series provides a stimulus for reflection before, during and after medical decision-making. The way this is achieved is to use the videos in undergraduate and/or post-graduate(UG/PG) medical educational or continuous professional development (CPD) programmes to support the cultivation and development of practical wisdom for groups of practitioners. For instance, in a CPD programme they could view the series and then debate in groups the virtues they observe and how it  relates to the current cases and dilemmas they are experiencing. The flexibility and adaptability of the resources means that the practitioners can add a virtue continuum of relevance to the situation. Thus, practitioners can add and remove virtues, move along the continua and integrate with the particularities of the decision-making process for the individual case.

### Limitations

Our study does not claim to have captured all the virtues required for good and wise clinical decision-making, or to be offering yet another deontological guide, to be followed by medical doctors. However, it does offer a contemporary moral debating resource for medical educators and practitioners in their peer groups to decide on the relevant virtues for their context and the case under consideration.

This study is focussed on one healthcare profession—doctors. Further research is therefore needed, with inter-professional/disciplinary teams and integrated care groups to understand what it means for them individually and as a collective to make ethically wise and good decisions for patients and their communities. This is especially relevant to patient groups where a wide group of different professions must come together to make the right decision at the right time and in the right place e.g. older people with frailty and people suffering from mental health issues. To this end the research team have already initiated new proposals that address this call and gap in current understanding of ethical healthcare decision-making.

## Conclusions

*Phronesis* and practice virtues are interdependent. *Phronesis* helps adapt the practice virtues that enable doctors to make the right decisions for *this* patient, and the wider community. We offer here a starting point of “collective practical wisdom” from a group of medics at all stages in their careers. In this regard, the moral debating resource described is a credible tool for introducing and cultivating the concept of *phronesis.* As well as complementing the knowledge possessed by qualified professionals, medical trainees do not have to wait until they are older to be purveyors of wisdom or wise decisions. Instead, the latter could start to learn some *phronesis* from the wider medical community at the start of their careers. The videos, and accompanying resources, can be used both as an in-action and post-action debriefing tool. Future practice, research and policy on medical decision-making should benefit from applying this non-prescriptive approach to addressing the health and well-being of patients and wider society. The tool and the virtue continua support the General Medical Council’s (GMC) ‘Generic Professional Capabilities Framework’ [61] and, from a policy perspective, the GMC’s ‘Outcomes for Graduates’, which lays down the professional and ethical responsibilities for its doctors [62]:9–10].

There is an understanding that “the aim of medical education is to develop doctors who are reflective, empathetic, trustworthy, committed to patient welfare and able to deal with complexity and uncertainty” [60]:431]. The 15 virtues in Table 2, interpreted from the narratives of our participants are the *in-situ* virtues that these UK practitioners conveyed as important to their practice. In medical ethics undergraduate, postgraduate and CPD programmes the resources described here can be used to examine and debate ethical dilemmas and challenges faced by actors in low, medium or high resource healthcare contexts and therefore are of international relevance.

## Supplementary Information

**Additional file 1**: Title-Final 15 Virtues Dataset. Description- the manuscript shows only 4 findings while, as stated in the manuscript, this supplementary file shows all the consolidated 15 virtues gleaned from the participant’s narratives along with participants’ quotes.

**Additional file 2**: Title-Virtues from observation data. Description- the file shows the virtues observed during observation of MTD, in tabulated form.

## Data Availability

The datasets generated and/analysed during the current study are publicly available at: https://www.birmingham.ac.uk/Documents/college-social-sciences/social-policy/phronesis/phronesis-in-medical-decision-making.pdf. The final dataset of the 15 virtues presented as the findings from this research are also available as supplementary files (1 and 2).

## Abbreviations

CPD: Continuous professional development
Exp.: Experienced
FY: Foundation year
GP: General practitioner
GMC: General Medical Council
MDT: Multidisciplinary team
NHS: National Health Service
Obs.: Observations
PMC: Phronesis and the Medical Community
PG: Postgraduate
REC: Research Ethics Committee
UG: Undergraduate
V.: Virtue
