# Supplementary material for: Using practical wisdom to facilitate ethical decision-making: a major empirical study of phronesis in the decision narratives of doctors
Source: BMC Med Ethics. 2021 Feb 18;22:16. doi: 10.1186/s12910-021-00581-y (PMC7890840; doi:10.1186/s12910-021-00581-y)
Supplement: Supplementary file 1 — Additional file 1: Title-Final 15 Virtues Dataset. Description- the manuscript shows only 4 findings while, as stated in the manuscript, this supplementary file shows all the consolidated 15 virtues gleaned from the participant’s narratives along with participants’ quotes. [file 12910_2021_581_MOESM1_ESM.doc]

# Final iteration of the consolidated virtues (Conroy, Hale et al. 2018)

1.Negotiation

Negotiator: Am I negotiating with the patient, their family/ carers and my team?

(Extremes: doctor decides; patient decides)

Most participants, across the cohorts, regarded negotiating with patients and others when making decisions about treatment or care as an essential virtue. The doctor’s role was seen as providing suitable and relevant information to enable patients /carers to come to a decision, but also to provide expert advice and guidance in the light of clinical facts, taking patients’ views into account, and thereby enabling informed choice in partnership with the patient.

*“I guess that would be my approach, just to seek out as many facts as I possibly could on the one hand, and for more… difficult decisions, just talking to the patient and trying to get to know them a bit better and their kind of particular outlook ….. and then possibly based on that, kind of guide them to a decision that I think might suit them better.” (B102).*

Experienced doctors spoke of the importance of a dialogue and how exchanging information resolves conflicts and enables patients to make an informed choice.

“*a constructive conversation both ways. I’ve got something to say but let’s not jump to a decision now, because that would be wrong.” (BX02).*

However, decisiveness was seen as being respected both by doctors in training and some patients they come into contact with, sometimes it was felt patients implicitly seek paternalistic guidance, as they may find decision-making a burden, or rely on the doctor’s expertise and knowledge to guide them

“*Sometimes people do respond well actually to someone taking control of the situation, even if it’s in a way that you would think would surely be completely inappropriate, but they [the patients] respond well to it.” (B112)*

*“[O]ften when you ask people, ‘What do you want to do? These are your options’, they just say, ‘You decide doctor. You’re the doctor, that’s why I’ve come to you.’” (BX03)*

‘*99.9%’* of patients would say: “*What do you think I should do?” (WX01).*

Sometimes persuading patients in their best interest, according to some participants, is necessary, patient autonomy notwithstanding, since:

*“[A] patient doesn’t understand the severity of the decision they’re making, and perhaps only when they’ve seen people who don’t have the procedure done or don’t have an operation might they learn… the actual nature of the decision they’re making, because we see it, whereas they don’t.” (WX02).*

At times this leads to doctors deciding, contextually, to refrain from full disclosure of relevant information to patients, justified on the basis of preventing further distress. However some considered that this could undermine trust between doctors and patients and lead to unwise or wrong decisions (see Trust ).

*“[H]ad to couch it a little bit and actually we’ve reduced the medications by ourselves …. what I couldn’t do at the time was say what could happen, because she’d have gone into meltdown.” (BX03)*

Some doctors, guided by the duty to respect patient autonomy, assume the role of information provider, enabling patients’ decisions to be implemented:

*“But, for me, a good decision is one where the patient is the one who essentially makes the decision, or puts forward their wishes, and we then, as the clinicians, allow that decision to come to fruition.” (B107)*

Others, however, see providing information and asking patients to decide as an abdication of a doctors’ duty. There was a sense that decision-making is a burden which should be taken up by doctors, rather than ‘imposed’ on patients:

*“Sometimes, I think we put…patients in a really difficult position. Are we doing shared decision-making, or are we saying ‘I’m offloading the responsibility to you and you make that decision.’?” (BX03)*

2.Justice/Fairness

Just/ fair: Am I being fair? Am I considering the wider aspects of who gets treated? (Extremes: all get treatment; only a select few get treatment)

There are many “pulls and pushes” that doctors experience in their practice, which impact upon their decisions. According to one experienced doctor:

*“I explain to the juniors, we work in a job where we are pushed by different forces as doctors... So as a decision-maker, we are pushed different ways; we have our organisations pulling from us, we have the patient, we have the family, we have our own knowledge. So these kinds of forces, they are pulling in different directions. Sometimes they are all in the same direction which is good and that makes it easy, but sometimes they are pulling in different directions…” (WX04)*

Access to lung cancer treatment for smokers was a difficult decision with justice and fairnesss implications discussed by a second year medical student while an experienced GP described the need to be more nuanced in applying fees so that patients’ ability to pay for certain services was taken into account.

In such circumstances being just and fair was considered an important virtue. Though the spectrum of opinion spreads from a select few getting treatment to everybody getting treated, most considered that fair and equitable distribution of treatment is important. As a result, they would consider whether submitting to a patient’s request for a particular treatment / investigation is a) necessary and b) fair to other patients. This is directly influenced by available resources, and its impact on their clinical practice. The aim then is for the doctor to reach a “*shared management*”:

*“[T]hat actually is suiting the patient a lot of the time …. but also being resource aware and also suiting the practice’s policies.” (*NX03)

Communicating with patients on the principle of fair allocation of resources is tricky. Some patients understand, while others find it difficult to accept if they are denied treatment. Patients may feel they are being “wronged” according to one new doctor:

*“Well yes, okay, you have to treat everyone fairly, fine. I can’t really argue against that. Ironically, the patients who never get there are the probably the ones…. who are very – like I was young and hot blooded, they feel like, ‘What do you mean? I don’t care if there’s budgets, I’m in front of you, I’m sick, you’re supposed to look after me’ or whatever.” (W104)*

3.Trustworthiness

Trustworthy: Am I maintaining confidentiality, am I trustworthy, am I behaving with integrity? Have I /did I display trust and integrity, maintain confidentiality?

(Extremes: overly trusting/ trusted; shows no trust in others)

Trust was considered a necessary virtue by many participants

*“I think we are in a profession where trust underlies almost everything we do ...numerous things that invade people’s privacy and invades their sense of self…and I think if there’s anything that undermines ….. just is abhorrent.” (B108)*

However, with practical experience, this became less clear-cut; because *“real-life situations are often a bit muddier and murkier than that.” (B506)*. Despite this murkiness, it was seen as important that doctors act with integrity and honesty in *“all parts of your life as well.” (BX03)*. Describing a patient who eventually died in prison, with underlying medical issues which had not been adequately followed up:

*“I think the challenge, if we go back to ethics and wisdom, has to be underpinned by the integrity of the practitioners …. wisdom must be underpinned by people demonstrating a high level of integrity, …I think this whole area of phronesis, applying wisdom is a dynamic process of continual learning that is underpinned by integrity, honesty and continually learning from those.” (BX06)*

Breaching confidentiality was a serious ethical dilemma, which could lead to patients’ loss of trust in the doctor, and therefore needed to be carefully weighed up. Confidentiality was challenged where doctors and other health care professionals held personal views which conflicted with the patient’s, or with the care plan they were following. Other doctors spoke of the need to maintain the confidentiality and potentially avoid social stigmatisation of patients by withholding information from partners or family, examples included (i) a young woman whose liver condition may have been exacerbated by the contraceptive pill, who did not want her mother to know, nursing staff advised the junior doctor to send the discharge letter direct to the GP, not home with the patient and her family; (ii) a patient who did not want to tell their partner their HIV status, and (iii) patients with mental health problems who did not want partners, family or other agencies involved. One participant felt that this would be ethically justifiable if knowing the truth would do more harm than good: *“…you owe them the truth but then you’re not telling a lie if they don’t ask.” (B208)*

Withholding information from patients could impact on both care at the time, and trust in the long term doctor – patient relationship.. Attitudes on this had changed over time, but it still remained an issue: one experienced GP told a story of a patient who was told after birth that she had and the practice “*had known about this for years* “ ( NX02), similarly :

*“Do I unintentionally cause her distress or confusion more so than she already is to get this message across?’….. ‘Should I actually tell her or leave her in the dark?’ That is quite a difficult decision to make.” (B201)*

*“..a lot of … doctors … kind of focus on ... making patients happy, whereas for me ...possibly more important than that is being honest with patients; and sometimes you can’t do the first if you’re going to do the second. […] on my last rotation I was in a respiratory clinic, and there had been a patient who had been on the ward, they’d kind of suspected that she had lung cancer and … a couple of people had kind of tried to have a conversation with her to … like broach the topic, and kind of felt that they’d pretty much told her and prepared her and then when she was actually told the news.. more explicitly she was completely taken aback and completely surprised by it, because people hadn’t been frank enough with her.” (W207)*

In addition to integrity, confidentiality and honesty, communication was also seen as a key enabler of trust and poor communication and lack, or absence, of rapport can lead to different choices by patients.

*“What helps trust is clear communication with patients and families, and with timelines which are kept to.” (BX05).*

An example was offered of a patient who did not go ahead with an important operation, because they felt not all options had been discussed and:

*“…the patient, I think, felt that that there wasn’t a good level of communication there; and I suppose that affected the trust that the patient had with the doctor.” (NX03)*

Trust in the doctor enabled patients to confide in them, and so was helpful in reaching an accurate diagnosis. Communication as an enabler of trust was allied to being seen as knowledgeable and authoritative.

*“My consultant at the moment is really good, and he’s very good at communicating with the patients and they all love him. He’s very authoritative, people trust him….. I think it’s good to have people trust your opinion. You don’t want to be someone who people think is going to make mistakes. You need to, I think, to be confident in your decisions and have the knowledge [and confidence?] to know that you are right.” (B109)*

Trust in colleagues was seen as an enabler of good health care to patients, and trust, team work and discretionary effort were seen as going hand in hand.

*“And if I’ve got assurance that they are doing that to the best of their ability, and I’ve got absolute confidence in my team, then they will do that as long as it’s physically possible. There is a point where actually they can’t do any more, but if you’ve got confidence in them…then you know that you’re on firm ground.” (BX05)*

*“…we absolutely got all of our routine work done, saw every other patient. We were able to utilise our resources, but also people were going that extra mile to try and get this patient coordinated, to get this family sorted…. and make sure we didn't miss the slot to get in…. keeping the relatives fully informed, aside of all the clinical technical bits that go with that.” (BX10)*

4.Lawful

Lawful: Am I acting lawfully? Is the goal lawful?

(Extremes: constant litigation worries; ignores legal constraints)

Acting lawfully was another important virtue that emerged quite early in the data. There are experiences where law and duty of care, though essentially compatible, may be irreconcilable:

“…*they might be more defensive in terms of investigating more, doing more tests and actually, that might not be the best approach. You might be doing harm to the patient by putting them through investigations in such a manner.*” (NX03)

‘Covering one’s back’ and the underlying threat of litigation were recurring findings. Awareness of these ‘legal pitfalls’ prompted doctors to practice defensive medicine since:

*“….the legal underlyingness to it I feel is always prevalent.” (B111)*

A drawback of being constantly obsessed about litigation is that at times, mistakes are not discussed and opportunities to learn are missed:

*“I think people try and brush them under the carpet a lot, and the problem is in the NHS there is quite a blame culture, ….. and when something goes wrong and things weren’t quite handled correctly, people get very defensive… because there is a lot of fear about, you know, losing your licence or being called up to disciplinary.” (W108)*

5.Being Collaborative / Seeking guidance

Collaborator: Am I referring to appropriate written guidance? Am I seeking guidance from my senior, my team, and my peers?

(Extremes: constantly seeks guidance from peers and/or professional bodies; wholly self-guided/does not consult)

Most participants felt the present day clinical paradigm is one where isolated decision-making is neither advisable, nor possible and that seeking to involve all those entrusted with a particular patient’s care allowed holistic decisions, tailored to the patient. Guidance is sought from peers, seniors, multidisciplinary teams, nurses and particular professional guidelines. This was corroborated by the project’s observations of different MDT meetings. When making decisions for complex cases, team members found that the progressive decisions reached and displayed on the whiteboards were useful, as *“they help prioritise and review decisions.” (Obs.1)*

Guidelines however, though useful, require contextual interpretation; and this contextual awareness can be shaped by others who know the patient, such as:

*“[T]he nursing staff who cared for the patient throughout, I relied on hugely because actually I was very upset by the whole situation and felt that the burden of the decision was on me. …and even the night sister … just made it more logical, and decision-making more logical. I do rely on my consultants for the ultimate decision quite a lot of the time.” (BX01)*

In observation 2 the roles of the Occupational Therapist (OT), Physiotherapist (PT) and speech therapist were seen to be central to certain patients’ treatment as they had the most information regarding them. The registrars and consultant relied on the OT and PT in particular to provide almost the whole of the summary of information. These collaborative discussions become critically important when making ‘deprivation of liberty’ decisions, and concluding that a patient lacks the mental capacity to make their own care decisions. The observation made it clear that:

*“The lead consultant would ask questions and appeared to be kind of taking it all in, cross-referencing information he got with his records on his computer. More often than not, he would defer to the decisions of the PT and OT…..The nurse had a lot of say as well about how patients were progressing towards their goals.” (Obs. 2)*

This though was not universal, at another MDT observation (Obs. 3), the discussions were mostly contained amongst the doctors; with barely any input from the rest of the staff.

Most medical students were of the view that it is far better for “not-so-experienced doctors” to defer to people with more experience:

*“[Y]ou know, bigger decisions, you’re not going to want to take that onto yourself, you’re going to defer to people that have got the experience.” (W203)*

Newly trained doctors find it easier (and safer) to seek guidance from and feel reassured by more experienced doctors, as observed (Obs. 3) in an Emergency Department environment, when the junior doctor requested a consultant to discuss ‘an older patient with complex health and social problems’ and an early career doctor:

*“…. they’ve probably made that before and they can tell you with experience the outcome and why. And they might come up with ideas as to why your idea might not be the best for that patient.” (W101)*

At the same time, experienced doctors also seek guidance in challenging cases and appreciate the advice given; for not only does it take the pressure off, it also helps make a better decision because:

*“…sometimes that consensus is really useful because you’re basically going through the arguments … and again clarifying some of the aspects of it, I think.” (BX11)*

However, if a doctor excessively seeks guidance, this may have the result of hindering their ability to make independent decisions. By continually deferring to others, they will fail to acquire the requisite professional experience to develop both this vital skill themselves as a medical practitioner, and as a result will tend to “*stall on patients because they’re unconfident.” (WX05)*

Another consultant was of similar view. After being approached time and again by a senior trainee, they eventually deliberately held back their advice and let the trainee decide independently; while at the same time remaining close enough to the decision to ensure that there was no compromise in patient safety:

*“[A senior trainee] came to me with this incredibly complex case, presented every detail of it to me, and then said, ‘Tell me what to do.’ And I refused. He got very angry with me. And I said, ‘Look, you know, in two months’ time, you are going to be the consultant.” (NX07)*

Although it is good that trainees have ample medical knowledge as well as knowledge of guidelines, it is the lack of practical knowledge which hinders wise decisions. Some participants therefore recommended that when doctors engage in interpreting guidelines, they do so contextually. This could mean referring to more experienced doctors to gain insights into wider interpretation of the guidelines in particular circumstances; or to ‘read between the lines’. After all, guidelines are designed only to guide practice:

“*…so we’ve got an SHO …. he has very good book knowledge, he’s very academic, he knows the guidelines for everything off by heart but he doesn’t really have a grasp of the fact that not every patient can be treated as per guidelines. And we’ve been trying to explain to him that a guideline is just that, it’s a guideline, it’s not rigid; it’s meant to guide your practice…..* *he’s had real issues with not calling for senior support because he feels that he’s got a guideline to follow and that he follows it..” (WX05)*

6.Cultural competence

Culturally Competent: Am I being culturally competent: am I considering the patient and their family/ carers’ values and beliefs as part of the establishing of the facts?

(Extremes: uses own values and beliefs; goes with patient’s values and beliefs only)

Respecting patients’ values and beliefs is a virtue which all the cohorts acknowledged as important. Many of the participants said that they consult their colleagues to understand cultural issues and to consider what the right way forward in a particular situation might be. Having said this, there were those who narrated experiences in which the doctor chose to follow their own beliefs and values, rather than those of the patient they were treating. A doctor who experienced a situation where one doctor refused to provide an intervention as it challenged their personal beliefs (and another colleague had to be approached – which inevitably lead to delay in delivering the treatment) considered that:

“*it is important* to *park your own values. You should not allow those values to affect the decision.” (BX04)*

A 5^th^ Year medical student narrated their experience of a consultation in a sexual health clinic, where the doctor seemed judgemental towards a patient, which took the participant by surprise:

*“I think he said something like, ‘Are you gay or straight?’ or something. Just, like, which is incorrectly phrased? There’s far more, like, tactful ways to do it. But he, kind of, shouted at them, so, ‘Are you gay?’ kind of thing.” (B501)*

Understanding cross-cultural issue was seen as important in building trust. Discussing the way in which rehabilitation can be seen to follow a ‘white Anglo-Saxon’ model, one experienced doctor explained:

*“The Muslim perspective is a more vitalist perspective in terms of – you can have everything that’s possible. Whereas I think there’s – generally there’s mistrust of the NHS that we pull out too soon, and we don’t do everything that’s possible, and we don’t do everything we should be doing etc. but that’s compounded by an ethical – a cultural view of life I think….. there is a cultural clash, so there is mistrust that can be on both sides. The only way to get around that is to recognise that there is a difference in view and maintain open dialogue.” (BX05)*

Most views aligned with what this experienced doctor who said:

*“[A] huge part in my decision-making is influenced by I think the patient’s values and beliefs, and the family’s values and beliefs as well.” (BX01)*

7.Emotional Intelligence (including interpersonal communicator)

**Good Communicator:** Am I displaying emotional intelligence? (Extremes: becomes too involved/ over emotional; distant/ aloof in communication)

Good interpersonal communication is another virtue considered commendable by our participants:

*“you can be the greatest doctor in the world but if you can’t communicate, nobody will do what you say, will they? (BX103).*

However, it is equally essential, that the doctor has the clinical knowledge regarding the disease at hand for:

*“you can be a very compassionate person but a useless doctor if you don’t know what you’re doing.” (W207).*

A few narrated experiences where there was an apparent lack of interpersonal communication skills displayed by a doctor:

*“…the clinician who saw her [the patient] wasn’t very communicative and reassuring in his approach to the patient… [the patient] was having a miscarriage, [the clinician] left it at that; left the room, and I was standing there with a very distraught couple… I told the clinician and he said, ‘Oh they’ll probably figure it out some way along the line’. And wasn’t very keen on going back and telling the patients – reassuring them****.****” (W502).*

The most commonly held position is where there seems to be an impasse or a clash between the beliefs of the patient and those of the healthcare team, it is important to avoid this escalating into mistrust, therefore:

*“The only way to get around that is to recognise that there is a difference in view and maintain open dialogue but be aware that however much you say in open dialogue, there may be a point at which you can’t get the message across any further, but where others coming in and saying the same things but from a different perspective, can be very valuable.”* (BX05)

This has lead more experienced doctors to give the following advice to junior doctors:

*“[W]henever you have a patient that is not straightforward …. a patient that has chronic issues, that has social issues, that is frail, that is elderly, then when you have that or somebody that has either mental health or personality issues, then one of the things that I will do is I sit down, I introduce myself to the patient and then my first question is, “How can I help you?”* (WX04)

This, the doctor considered important; because according to him this is how patients become amenable to discussion, and provide information which gives new insights into their illness, and helps make holistic treatment plans. This requires empathetic communication:

*“I think you have to, I suppose, temper your objective, rational facts for your decision-making process in a way that comes across as empathetic and sympathetic and looking at a bigger picture view beyond the current situation; and also to help parents to think about things from their baby's view.” (BX12).*

8. Aware of limits to treatment

**Recognise treatment limits:** Am I recognising limits to treatment – what is the end goal here? (Extremes: treats at all costs, Deficiency; shows limited consideration of treatment options)

Taking the whole picture into consideration before making decisions also requires doctors to realize the limits of their treatment decisions. Most thought that recognizing when a treatment is no longer likely to be beneficial is not only important because of resource limitations, but more importantly it can avoid unnecessary treatment which is undoubtedly in the patient’s best interest. There are doctors who would treat patients at all costs, because of the pressure they are put under by the family:

“*so they [family]wanted absolutely everything done for her; they wanted a bridled NG and she pulled out two or three NG tubes…... so all you were left with was the family demanding unrealistic things.” ( N103).*

There are some instances when doctors themselves are keen to continue treatment either because ‘not treating’ does not sit well with them, or through fear of litigation / complaints; i.e. practicing defensive medicine:

*“[T]here are some consultants that don’t like to put people on end of life. It just doesn’t sit well with them. They don’t like to deal with palliative care and end of life, because their idea is they want to fix.” (W101)*

By contrast, , there were a few episodes narrated to the research team in which doctors did not discuss with the patient or family all the treatment options that were available. The participants were of the view that this would lead to erosion of trust in the medical profession, something that would not benefit them, or society:

*“The patient did say that they felt that not all options were discussed at that opportunity; because the patient, who enjoys reading extensively about their health, looked at lots of things online and thought that there might have been lots of other options to be discussed that weren’t. And so…I suppose that affected the trust that the patient had with the doctor.” (NX03)*

However most were of the view that it is important to realise that when the harm caused to a patient by a treatment is exceeding the benefits it was intended to provide, and in line with the best interest principle, that treatment ought to stop. Describing an episode involving a patient with hypoxic brain injury following a cardiac arrest, a doctor considered that the cardiologist’s recommended medication, although keeping the heart well vascularised, was compromising the patient’s quality of life and so thought:

*“[T]his is a man with a poor neurological prognosis where if he hasn’t got blood pressure that’s perfusing his brain, that might actually impact on his alertness and stuff; and so there may come a point where we actually have to say to the cardiologist, ‘Actually, we’re stopping some of your medication. We accept it may… result in a shorter lifespan, but if it’s something that’s going to keep his blood pressure at a level that keeps him cerebrally perfused and able to function then he’s got quality’. That would have to be a ‘best interests’ discussion.” (BX05).*

9 Mentorship.

**Approachable Mentor:**  Do I have a mentor I can approach? Am I approachable as a mentor? (Extremes: constant mentoring / overly directive; no interest in mentorship/ being approached by others)

Participants described both negative and positive experiences of mentors The importance of mentoring, role models and structures which enabled and facilitated good mentorship were discussed . A good mentor was variously described as approachable, someone who did not provide solutions but who asked questions, and built the confidence of others. Poor role models were absent and/or unapproachable. There was a perceived impact of mentoring on the quality and safety of care: good mentors demonstrated and ensured that conversations with patients were more inclusive, and that proposed treatment was safe and appropriate. The experience of less approachable mentors was that juniors did not always check or get reassurance that their approach was the most effective in that situation, for that patient. This could impact directly on patient care because:

*“if you are not approachable and the junior is scared of asking because everything he has received so far is bad temper, then your knowledge is not passed on.” (WX02)*

*“I think they were very stressed, and when I went to them about not knowing what to do with a patient, they, almost, shot me down, and I felt then, I couldn’t go back to them about it.” (W101, Follow-up) and this could mean that one is :”less likely to call them if you’ve got a problem, which is a bad thing, less likely to I suppose… well, you just don’t enjoy your job as much when you’re with those sorts of seniors.” (B110, Follow-up)*.

Thus a good mentor was described as someone who is approachable :

*“So, she is really, really empathetic …..She made time to listen to what actually mattered to the patient, and often that wasn’t how soon they could have the complex surgery that they needed, but when their partner could visit, or [something] really simple….She was always approachable, but also, I kind of learnt that to be empathetic and caring, you don’t have to be a pushover.” (BX07).*

Not only is an approachable mentor available but also listened and questioned as opposed to being directive and providing immediate answers,with safety netted where needed. Discussing a patient on their operating list with a perforation:

*“So, I think the first part in allowing people to be wise is to make sure that it’s not a one way flow of information, that actually the more we talk to each other, both peers and senior colleagues, the more we talk about it, the more we allow ourselves to explore the areas that we’re weak at and to recognise our own weaknesses and our own thought processes, that we can all become wiser.” (WX09)*

Some senior doctors were described as aloof or unapproachable, or simply*: “rude and quite obnoxious” (W104) and “not wanting to make themselves available… would come across as brash and disinterested in wanting to move things forward” (NX03).*

*“…if it is a closed-door policy, even a junior struggling with ethical decisions, why would they turn to their seniors?” (N507)*

It was also emphasised that this was a two-way relationship; mentees being willing to ask for help and mentors willing to explore a decision.

*“…it's maybe not decision by decision that needs to be covered, it’s that moral compass and willingness to explore options and discuss and talk to other people about it and confer, that’s I think probably where the education needs to be; “it’s the willingness to ask for help and to talk things through.” (B504)*

Mentors also attempted to ensure a blame-free environment , an experienced doctor described their approach:

*” Because one mistake that a lot of our colleagues do, because it’s the way we were trained, is being very hard…told off…..that really drops the confidence of anybody to the floor… The best thing is if there has been a mistake or if there has been a wrong decision, just help them to see that by themselves… to analyse their own actions and their own thinking process. And then what I normally do is try to offer them the view of, ‘Have you talked to the patient? Have you asked the patient? What do you think is best for the patient? What do you think that the patient wants?’ And trying to force them to put on the shoes of the patient or the family, ..,flex the guidelines without being in a risky area. So, to what extent you can be flexing the guidelines and still not be putting the patient at risk.” (WX04)*

An Emergency Department consultant’s experience and intuition made him suggest not discharging a patient from ED who went on to show a full blown myocardial infarction on his electrocardiogram. But he couldn’t explain why he had suggested to the junior doctor not to send him home:

*“.. sometimes it’s clear, and certainly one thing I try to do when I’m on the shop floor and have a case presented, is I try to help people develop their own decision making process by trying to get them to think about what they would do, why they would do it and why they wouldn’t do something else, rather than just saying, ‘What are you going to do?’ But there is still that bit of learned instinct and it’s how you fit that together with what you can teach people. And then there’s part of experience I think that experience gives you that’s difficult to teach, but you can’t turn somebody who hasn’t seen enough cases into someone who has the experience to make accurate decisions.” (WX03).*

An approachable mentor was described as available but someone who listened and questioned as opposed to being directive and providing immediate answers, but who also safety netted where needed. Discussing a patient on their operating list with a perforation:

*. So, I think quite commonly you’ll get medical students or junior colleagues that are almost – that will present a case to you and expect you to give them an answer, rather than get them to work through it : ‘Well talk to me about it, talk to me about what you think it might be …’. So, I think the first part in allowing people to be wise is to make sure that it’s not a one way flow of information, that actually the more we talk to each other, both peers and senior colleagues, the more we talk about it, the more we allow ourselves to explore the areas that we’re weak at and to recognise our own weaknesses and our own thought processes, that we can all become wiser.” (WX09)*

At the end there was a balance to be struck between mentor and mentee which partly depended on the level of confidence of the mentee, as this might determine the safety of care provided:

*“…you do have a little bit of confidence given by your seniors to do things, so they tend not to scrutinise your actions that much, which is a good thing; but it can also be a bad thing, because you can gain too much confidence in yourself.” (WFY203)*

Providing effective mentoring takes time as explained by one early career doctor:

*“I think one of the problems with some of my bosses and seniors is …they don’t communicate their thought process to the juniors; so they will just say ‘This is what we are doing, of course he shouldn’t have this.’ and you never get into why, and the better teachers are the ones who will take a bit of time to… it’s so helpful when you have those seniors who are… who voice what they’re thinking and alk to the team.” (B104) and (B104 Follow-up)*

Shadowing and observing senior doctors was seen as helping not only diagnostic and other clinical skills, but also communication skills:

*“I think shadowing is important. Trying to learn something in this session that might be transferable to other areas… try to see how I do things, how I interact with other people and then try to put yourself in my shoes. If you were me, how would you have made that decision?.....I think there are roles for role models, but I think human beings all have flaws, so therefore my experience tells me that it would be a myth if I rely on one person as a role model, or rely on one person for everything.” (NX05)*

In addition to the attributes of mentors, structural issues were seen as impacting on mentoring and included factors such as doctor numbers, and changes in the organisation of medical training meaning less time and less emotional investment in mentoring:

*“I think the pressures from the seniors are fed down now more to the junior tier… as the senior doctors tend to be under more pressure they are probably less able to support the juniors and nurture them, care for them, than perhaps in the past. It doesn’t help that our juniors come through for a four month block now, whereas it was six months when I was training and I think that makes a difference because …Well I’ll just get them up to speed and then they’ll be gone,’ which obviously is a big loss to those individuals and to us” (WX03)*

Along with the restructuring of medical education, a shift to a more consultant-led service and an overall more risk-averse approach has also reduced practical clinical experience for doctors in training:

*“As I’ve gone through my career, some simple things like stitching up an episiotomy I did as a medical student, and they used to call us as medical students when the lady you’d delivered, come do the stitching and then that stopped because it wasn’t safe and it went on to being the SHO, the next grade up that was called. And now really even some of the Registrars will come and ask us to watch them stitching up a difficult episiotomy. So, it’s drifting towards much more of a consultant delivered service rather than … a consultant led service (BX13).*

10.Balanced approach

**Holistic:** Am I taking a Holistic approach, am I considering all facts which are known about the patient not just the concrete scientific facts? (Extremes: tries to cater to all aspects; pursues just one approach e.g. science only)

Keeping a balance between medical and social needs is an important virtue to many of the participants and a holistic approach is required where doctors bring the practical wisdom, technical and communication skills gained from their past experiences to bear, in order to:

“*[A]djust my skillset to the different patient and depending on the presentation and depending on the characteristics of the patient, bear on the case at hand and make a decision…. I would say that is what my experience has given to me. It’s not only the knowledge; I have got the knowledge, but the knowledge is one of the skills [along with] the communication, empathy and others.” (WX04)*

In the context of seriously ill children, decision-making in conjunction with families was seen as being enabled when doctors are able to:

*“…temper your objective, rational facts for your decision-making process in a way that comes across as empathetic and sympathetic, and looking at a bigger picture view beyond the current situation; and also to help parents to think about things from their baby's view.” (BX12)*

Although there are instances where, the decisions made are *“more formulaic and less wise”* (NFY201) generally the approach needs to be more *“a list of needs rather than a checklist of jobs”*. (NFY 201) One experienced doctor stated this explicitly:

*“…we have a holistic view of the whole person, so they’re not just a heart that’s been damaged with the rest of the body attached to it; we’ve got to look at the whole picture.” (BX05)*

Some find that the holistic *management* of a patient with an illness, with all its attendant social, emotional and multi- disciplinary and agency aspects, is more difficult than simply *treating* the patient’s physical illness on its own:

*“Yes, yes because it is this limitation of ways, it is this difficulty of managing an illness rather than treatment of an illness which is the more interesting bit, the more difficult bit and there are never going to be mathematically accurate answers.” (WX06)*

Some of the doctors progressing through their early training reflected on their ability to have evolved from making clinical decisions informed by their clinical knowledge, and guidance from either senior doctors or specific guidelines, to making holistic care decisions. This approach is underpinned by their enhanced ability to gather a *“deep understanding of all of the patient’s needs”* … that *certainly strengthened when I finished my F1 year.” (NFY2-01)*

11.Reflexivity

**Reflexivity:**  Am I being reflective? Have I given myself time to reflect on this decision? (Extremes: overly analytical towards his/ her own actions (naval gazing); never reflects on actions/ decisions)

Reflective practice was generally seen as enabling learning and the development of skills in decision-making. Some focused on the formal mechanisms which enabled reflective practice such as reflective diaries, GP appraisals, significant event analysis and debriefing sessions; while others described less formal sharing of stories. All of these formal and informal reflective processes are about reflection ‘on-action’, whereas doctors and medical students also spoke about their reflection ‘in-action’ (reflexive practice) and how this was developing and enabling them to make wise decisions in the moment.

The benefits of reflective and reflexive practice were summed up as:

*“I think reflecting on practice is essential… I think it’s important in terms of wise decisions, that people realise the basis of wise decisions reflecting in and on practice. I think it’s also right that we would tell them… reflect back on what you’d done, your contribution, and how you managed it, including the painful… ‘actually it’s something I can incorporate in’.” (BX02)*

Medical students discussed various examples of reflection after an event/ ‘on action’ and one spoke about the process of reflection itself:

*“Yes, definitely. I think there’s a lot of reflecting. You have to reflect for our e-portfolios, ..thrilling.., but you do a lot of internal reflection as well. Every time I see a senior and see how they’ve made a decision, there are some ones where I think ‘Yes, I get why they’ve made that decision’, and then a few times, luckily not as many times, I’m like, ‘Oh, no, that’s a bit of a dodgy decision’…. you pick it up and you think, ‘Well, would I have done that myself?’.” (W209)*

Reflection was seen as a key part of learning:

*“I think often, as with many jobs you’re very pressured at work and you just sort of get through the day and get to the end of the day, and unless you have the time and the will to think about things then and go on and sort of seek ways to improve your learning and your own decision making or wiseness then you won’t improve unless you want to… and it’s trying to learn why it went wrong, and what could be done better.” (WX11)*

The way in which reflection can be used in a positive way to build practice, rather than leave lingering concerns, was described by both an experienced and a foundation year doctor:

*“…you need to be humble enough to review it, to revisit it and to say, ‘Okay I may have been doing it wrong, let’s review again, let’s review what happened, let’s review my own process, how I made that decision’, and if, after that review you get to the point that you think that you did the best decision, then you need to hold and you need to defend that decision. Because at the end, we are the advocates for the patient, especially if the patient has no capacity.” (WX04)*

*“We definitely share stories and it’s probably one of the main ways we either relieve stress or just vent frustrations. And, yeah, a lot of the times can be for advice or for just support, because everyone makes mistakes… speaking to people really does help, because otherwise you would probably end up quite depressed and probably quite neurotic in some ways because you can’t be hyper-vigilant all the time; you’ll second-guess yourself and that way you will probably end up doing more harm in some ways...” (B103).*

Reflection in-action, or reflexivity, was seen as a continual and informal process of development:

*“I guess mechanisms in place to self-check and self-regulate yourself and maybe that is part of being wise. Like that kind of self-reflection on the spot to say, ‘Have I completed everything I need to do?’ and to take the time.” (W101)*

*“…once you notice the dilemma or the situation, I think you should already be reflecting on it. It doesn’t have to be in a formal way, reflection is essential to developing as a clinician; so even if it’s not going to change the decision you’ve already made, in some cases it might.” (B108)*

For some there was a concern that debriefs and reviews did not have any lasting impact, or a conflation of these processes with investigations and the apportioning of responsibility or blame. These doctors were on the whole negative about the process of reflective practice, or the feasibility of finding or making time for it. The term ‘navel-gazing’ was mentioned in two interviews where doctors were reflecting on the value placed on decisiveness; and where reflecting ‘on-action’ was seen as prevarication:

*“…in a way, I respected his decisiveness for taking a practical course of action and moving things on rather than perhaps [taking] a slightly more navel-gazing approach.” (B108)*

There was a sense that this attitude was slowly changing, but some doctors were still aware that they may be labelled as ‘navel-gazing’ for taking time to reflect on a decision:

*“I think because I am a little ‘navel-gazing’ and a bit reflective generally, I think I understand a lot of my processes of how I make decisions. Also how sometimes I might procrastinate about a decision.” (BX03)*

The pressures of work were seen as detracting from reflective practice. A diary entry highlighted the importance of reflection, but indicated that ward pressures meant there was limited time for reflection: the diarist felt the focus was on learning from mistakes and avoiding investigations. Another diary entry discussed the care of an 80 year old woman admitted to hospital with sepsis of unknown origin, where medical opinion differed, and the doctor had to *“remain objective and not judgmental, and this does require reflective thinking* ..t*he fast pace of patient care in acute medicine, high demand, constraint of capacity and even competencies does put strain on the services of medical providers. Reflective practices become difficult. This may easily translate into deterioration of services and poor patient outcomes.” (WX06)*

Speaking about consultants who did not consult or reflect:

*“I think it is a bad doctor that goes and makes rash decisions and is very confident about what they are doing, however much experience they have because I have seen consultants make rather dubious decisions and be very confident and forthright with it and because they are consultants everyone respects their decision and ultimately it is their responsibility….but the really good consultants …it is that knowing your own limitations…that reflective practice and there is a recognition of that in medicine but it doesn’t mean everybody does it.” (W108)*

12 Courage (to speak out / have difficult conversations)

**Courageous:** Am I having any necessary difficult conversations with the patient/customer, their family/ carers and/or with my peers, my seniors, management? (Extremes: takes foolhardy risks; cowardice/avoids conflict)

Having courage to speak out or the confidence to question was seen by most medical students and doctors as related to their own experience, and therefore confidence in their practice. This enables doctors to challenge when they are being asked to do things they consider unsafe or unethical, for example being asked to sign laboratory request forms for intra-operative tissue specimens which had not been labelled at the time they were taken:

*“If I was a little bit less confident in my practice, I may have gone along with the registrar -that’s a scenario where confidence is putting your foot down.” (B105, Follow-up)*

*“… but I think at least if I’d been younger, I might have been more easily coerced into actions or making decisions that I wasn’t entirely comfortable with.” (N104)*

Support of colleagues helped doctors and medical students find courage. One foundation year doctor discussed a consultant who was very bold, often stopped active treatment comparatively early and did not communicate well with families or other staff. A group of junior doctors took the decision in this instance to “step out of line”, and escalate this issue because of their concerns about safety, and the poor working environment (B109, follow-up). Another spoke of the support of senior nurses when they had the moral courage to call in the critical care outreach team, to assess a deteriorating patient they were worried about, which went against the expressed instructions of their consultant and registrar (W102).

For some, there was also a need to learn appropriate ways to speak up or question decision-making and to ‘get around the culture’. This learning enabled the virtue of courage to be put into practice:

*“…most of the time, especially on this ward, I just say to the consultant ‘This is what I think, do you agree?’ Or, ‘Why are you doing that, I don’t understand why we’re changing this?’ And like they’ll usually explain which is fine so I don’t feel bad about challenging them. I wouldn’t say I don’t think they’re wise by changing it, I just want to know why they’re doing it. Most of the time it’s not disagreement, it’s just [that] I don’t understand.” (N106)*

*“…with me, it’s always if I think something is going to really detrimentally affect a patient’s safety, then I will speak up. But I’ll do it more in private, and I tend to phrase it more as a discussion, so ‘Why are we doing it this way?’ ‘What do you think of this instead?’ Or, ‘Would we not, perhaps, do this?’ I’ll let them*

*explain to me then, why we wouldn’t.” (WFY205)*

Tradition, hierarchy and power were all external factors which made it hard for doctors or medical students to raise concerns, or to challenge; and therefore meant doctors ended up avoiding conflict.

The medical hierarchy, along with experience, played a part in determining whether or how a more junior doctor raised an issue. It was part of that sense that seniority brings experience and therefore that senior doctors know best. A foundation year doctor spoke of a decision to amputate a patient’s legs to extend her life, and the registrar not agreeing with this decision: *“but it was interesting that he didn’t really raise that with the consultant.” (B104, follow-up)*.

*“I think a lot of people will follow the example of seniors, and do it – ‘Oh, well, they’ve done it, so it’s okay to do it’… effectively sheep. If you’ve seen someone do it, or someone’s told you to do it, and they’ve told you to do it, and therefore it’s okay, you maybe will do things without questioning them; whereas that’s dangerous.” (B506)*

Junior doctors talked of patients who ‘weren’t quite right’, and trying to escalate, but being told “no it’s fine, crack on”. One junior doctor reflected:

*“…should I have pushed harder at that point? When you’re junior, you don’t have much experience, you don’t know whether you were the important cog in the wheel or it would have happened anyway.” (B104, follow-up)*

A medical student talked of operating theatre ‘camaraderie’, which extended to making inappropriate comments about a patient:

*“as a medical student, sometimes you can feel very out place and unwelcome in those environments, and obviously sticking your head above the parapet could be seen as risk. The first step or action that you should always take is to try and correct it in the immediate like vicinity… if you can just say to someone, ‘Excuse me, I don’t think those comments are appropriate.’ Obviously, whistleblowing and maintaining patient safety is our priority, and people will be protected if they do that; but I think as a student, you could quite easily be sort of kicked out of the theatre or not allowed in and then be able to justify it for other reasons than having said ‘Oh, he’s a snitch.’” (B507)*

Individual decisions regarding speaking out were also seen as being affected by the seriousness of the issue, or the concern of speaking out against, attitude and approachability of more senior doctors. A good working relationship with more senior colleagues was seen as making it easier to challenge them or raise concerns, in that it does not require the same degree of moral or physical courage:

*“So yeah, maybe the relationship between doctors and students, and doctors and doctors, makes you more vocal.” (B503)*

The seriousness of the situation, and the potential for causing harm or distress to the patient or family, were all noted as factors in determining whether junior doctors or medical students found the courage to voice their concerns. A fifth year student spoke of their disquiet when a patient became distressed about a possible diagnosis of cancer:

*“…basically the patient asked, ‘Do you think it’s cancer?’ and the consultant very flippantly said, ‘Yeah, maybe.’ and just left. And, I was just like, how can you… ethically how can you do that? So, the poor patient was there, really upset, so I went and said to the consultant, it was really difficult because obviously at that point I was a CT1 student, I just said, ‘Oh, I’m just a bit worried, the patient looked really upset when you left, have you got any chance to go and maybe talk to them in a bit more detail?’ and they were like, ‘Oh, no, I’m busy on the ward round.’ and I was, ‘Well, I think they do look genuinely really upset and if you think about what you have just said, you have maybe said to someone that they have got a really serious disease.’ And… eventually they came round and they took, I think one of the nurses with them as well, and talked to the patient. I found it really hard, I did have another medical student who was on the firm with me, so I just felt better, even just having someone standing next to me, to be like, ‘I don’t think this is right’… a lot of us will tend to kind of try and bounce it to the F1 who will end up going up that way.” (N508)*

Concerns regarding the benefits of treatment, and being able to challenge and ask *“what are we trying to achieve here?” (WFY202)*, was seen as a particularly contentious area; for example the benefit of catheterising a dying patient (N108). Discussing a patient with a ‘Do Not Attempt Resuscitation’ order in place who was admitted to hospital, a foundation year 1 doctor tried to challenge the consultant’s request to take bloods and cannulate her to deliver antibiotics:

*“… having to have other staff members try and kind of hold her arm down… a very distressing situation. But it’s hard, because it was left for me to do it and it was an order from a senior. I felt it was unfair on the patient… I just did one attempt, and then I wasn’t going to do it again, cause I didn’t want to cause her distress;, I kind of said to him, ‘I don’t feel comfortable doing this.’ And he was like, ‘Fine.’ And he said, ‘I’ll do it’. So, he did it… then I was still unhappy about the situation because the family was becoming upset. So, I then found the Registrar, eventually. I think, with the Registrar’s input, the consultant agreed… not to do any more.” (W101, Follow-up)*

There was also a certain perception of those who ‘break the rules’, in that those who do challenge decisions or instructions are labelled as ‘mavericks’:

*“… this was one of the first cannulas I did, and I was sort of basically instructed to do it and I shouldn’t. I didn’t do it. I started to do it and I knew at the time that I shouldn’t be doing it, and in any case the cannula wasn’t in properly anyway so it didn’t work, so then the person who was with me sort of took over and the procedure was sort of botched anyway. What I should have said at the beginning of that was ‘I’m not happy to even attempt to do that’, and that might have then ended up the situation being different because then no attempt might have been made and then this patient actually might not have been made uncomfortable and all of those things… but I definitely knew I shouldn’t have done it. I’ve always held onto that, because I felt bad about itAnd I definitely think that that’s just part of my personality, in a sense, and that other people are more... you know, they’re sort of, I don’t know, mavericks or they’re less risk averse perhaps.” (N509).*

13 Resilience

**Resilient:** Am I considering my own resilience in reaching and enacting this decision? (Extremes: think you are bullet proof; avoids any stress)

Those who mentioned resilience by name spoke of the importance of being aware of their own emotions, and how they may impact on decision-making:

“… *and I have got that personal tendency that I'm a bit more black and white if I'm significantly stressed. So I think that you've got to have some kind of understanding that you've got your own limits to what you can deal with. And I've had cases in the past, and I know we should be withdrawing treatment on this patient tonight, but I've already talked to three other families today about this and I really can’t do another one. And actually going, you know, ‘we'll leave it to tomorrow. They're not in any pain. They're not distressed. I know we could do it tonight, but that's enough now’. So you've got your personal insight, yeah.” (BX11)*

Examples were given of those whose resilience was seen as low, and the impact of this on their decision-making, but very few examples emerged of those seen to be at the opposite pole in the sense of being immune to the stress; or ‘bullet proof’. It was felt that when resilience is low and doctors are burnt out, it is harder to make decisions or to have difficult conversations, and in these situations doctors become avoidant. Those seen as having low resilience were described as avoidant, being unable to make a decision and attempting to defer, and this can in turn lead to poorer decisions and subsequent patient outcomes.

*“Well enthusiasm, you have got to be enthusiastic about what you do, and if you are burnt out, again it comes back to first principles, because you can’t work at full pressure indefinitely.” (NX02).*

Junior doctors often spoke of being left feeling uncomfortable with patients and families if they were upset or confused, after having been seen by a senior doctor, and what to do in that situation. One spoke of a patient miscarrying, and the consultant not wanting to go back to see her:

*“‘Oh, they’ll probably figure it out some way along the line’. And ( the consultant ) wasn’t very keen on going back and telling the patients, reassuring them that sexual intercourse isn’t a cause of miscarriage.“ (W502)*

Conversely, doctors spoke of good will and ‘going the extra mile’ and the resilience this depended upon; but acknowledged that it does take its toll::

*“I think that is tough; you have to become quite mentally tough to park things, and offload them at an appropriate time, because otherwise it starts affecting your judgement and decision-making for the next patient. For example, on Friday, where the paediatrician had been on call the whole week, starting at 8.30am and finishing at 8.30pm, and by Friday evening, he was absolutely shattered – and actually, there was a massive queue in ED, but he said, ‘I’m too tired to make good decisions about this. I’m going to have to hand over to the night staff, because I can’t do them justice.’ He said, ‘I’m going to have to go.’ so he handed over, and let someone fresh have a go, and start fresh, because otherwise... you often feel like you want to help people, and you should want to stay and put in the extra hours, when they’re very vulnerable people and you want to do the best of them; and that knowledge of, ‘Actually, I’m not going to do a good job here,’ and being able to step back and say, ‘Now, someone else is going to do this job, just as well as I can - probably better, the state I’m in,’ I think that’s quite good ethical practice.” (B506)*

In addition to personal coping mechanisms, other enablers of resilience were seen as being team support and communication. Without these, personal resilience suffers. Doctors talked of difficult decisions which had not worked out well. Patient complaints, for instance, and times when they had been overruled on a treatment plan were described as hard to process and needing resilience on their part; but that resilience also stems from being sure at the time that you are doing the best for the patient. One doctor saw that the best way to cope with these difficult times was feeling valued in local teams:

*“… that recognition, that camaraderie within a team, I think, is much more important. That's what builds your resilience.” (BX10)*

The example this doctor gave was of the co-ordination and ‘extra mile’ required to deal with a difficult road traffic accident, including the high risk transfer of one patient to another hospital:

*“So trying to coordinate that sort of activity I think I was in for 16 hours that day… I think that's when the healthcare system especially just comes really into focus and everything happens. And that's the bit that sometimes people don't see.” (BX10)*

Stressful situations may not be recognised as such at the time; recognition of stress and its impact on the individual can come later, and so it is important to review and pick this up later especially decisions regarding whether or not to continue treatment; and spoke of the feeling hour-by-hour of not knowing if the baby will live - *“it’s too much adrenaline really.” (BX12).*

The constant pressures and different demands meant doctors being pushed and pulled in different directions. According to an experienced doctor, described a patient who could not speak but who they managed to communicate with and to establish that they had capacity and did not wish to have their PEG tube re-inserted. For this doctor a trusted coping mechanism was to keep the patient in the centre:

*“… as a decision-maker, we are pushed different ways; we have our organisations pulling from us, we have the patient, we have the family, we have our own knowledge. So these kinds of forces, they are pulling in different directions. I always go first for the patient, and I have always gone first for the patient. Sometimes we don’t talk enough to our patients… (WX04)*

14. Resource awareness

**Resource Aware**: Am I considering the resource implications? (Including but above and beyond any financial implications) (Extremes: obsessed with finances/ resources; no consideration of finite resources)

Linked to the virtue of fair distribution of finite resources, according to most interviewees was the issue of resource constraints. Some were critical of wasting resources for reasons other than the best interest of the patient. One reason was fear of litigation promoting a culture of defensive medicine. Narrating an episode where the consultant shifted an elderly patient to Intensive Therapy Unit (ITU) for non-medical reasons, this participant thought:

*“He [the consultant] then overrode that [Do Not Attempt Resuscitation Order] and put that she was for resuscitation… and kept her on ITU until all the family could arrive and say goodbye to her and then we could switch everything off… I think he was trying to take the least litigious route that ‘This family are going to complain afterwards, and that’s going to be a pain to deal with.’ The outcome was never going to be good. Resource-wise, people shouldn’t go on to ITU for that reason.” (BX03)*

, An overwhelming worry was *“the legal side of things”* and so even if it meant getting some ‘extra’ interventions completed, or referrals made,it would be best:

*“… because at least you might waste resources, but you’ve covered your own back, so justice says it’s not right but you’ve got to account for your own career and that kind of thing, haven’t you, and you don’t want to bring the patient to that unnecessary harm.” (W503)*

Similar sentiments but for different reasons were expressed by experienced doctors They regretted being placed in situations where clinical care is overruled by resource constraints:

*“[S]ometimes we are sort of forced to make decisions for the patient based on the resources available, and I think that clinicians should not be in the situation to make those decisions based on the resources… the managers and everybody will say – and the intensivist will say, “No, I think we have used a lot of the resources here, and we are not going anywhere, so I think we have to pull the plug here (BX04).*

*“Well actually I’ve only got a limited number of beds, so I’m immediately rationed by what I can do.” (WX09)*

Some doctors felt that although it is wise to be financially / resource-prudent, there may be times when doctors are pressurised into getting some tests done so as to expedite consultation, or allay their patient’s anxiety:

*“[H]e’s come back and back and back and just won’t accept the advice he’s been given, so the temptation is that you end up trying to give him something that he shouldn’t be having or doesn’t need because you just want to get the consultation finished.” (W105 FP)*

The degree to which cost and other resource considerations impact on decision making may differ with seniority:

“[T]*there’s a lot of pressure on treatment decisions to be financially cost-effective, but if I thought the treatment was the right thing, I think that decision for me would be made independent of cost or resource. I think that’s probably more of a big deal for more experienced doctors, to be honest.” (NFY2-01)*

Time constraint is another factor that participants complain leaves them ‘firefighting’:

*“I think we are so pressurised, time-wise and understaffed, that you end up kind of firefighting every day.” (WX03)*

Time allocation for each patient varies sometimes, depending on whether they are a junior or a senior doctor. Simply having the time to explain things to patients, thus having the ability to make processes *“streamlined in terms of time efficiency” (NX03)* is important.

15. Phronesis

The development of practical wisdom was seen by most interviewees as a process of time and experience. One medical student termed it *“learned experience”* in conveying information and reaching decisions with patients (N203), while a foundation year doctor spoke of it as being a mix of nurture and nature (B104, Follow-up) and for an experienced doctor:

*“…some people are inherently wiser, they are really wise people…now, whether that wisdom is inherent or … is simply because that person has walked past that journey ahead of me.” (NX05)*

Experience can, however, lead to ingrained negative behaviours and an assumption that you know it all. A foundation year doctor recounted a difficult birth where there was no pain relief for the mother, and the consultant seemed to show a lack of compassion for the mother in focusing on getting the baby out alive. They concluded that selection of candidates for medical school was critical, and:

*“… experience makes you better at making clinical decisions… but not necessarily in terms of ethical decisions… a lot of people get stuck in their ways.” (B504)*

Assuming that you know it all and following a text book approach can cause a doctor to be caught out:

*“You can’t make a decision based on what the textbooks say… because if the textbooks say it, you can only say that that’s right 99% of the time. There will always be the one case that will catch you out if you treat everybody the same… there’s things that are really rare, but they still happen.” (WX02)*

An experienced doctor highlighted another risk that arises with experience and seniority and was “*arrogant or foolhardy.” (BX04).* :

Similarly, another experienced doctor reflected on a senior consultant who regularly over- ruled on the basis of experience rather than knowledge*:*

*“Because evidence-based medicine tells* *you something else, but the experience of this doctor was something different, so there* i*s, kind of, a clash between the two, rather than both going forward in a symbiotic* *relationship…. Which is why I'm wary of saying that wisdom is the most important thing “ (NX04)*

For some medical students, *phronesis* seemed to be narrowly defined as diagnostic skills as opposed to the broader process of decision-making (as described by foundation and experienced doctors):

*“You know you learn by example, by following what someone else is doing… the art and the science of medicine… you need the clinical knowledge and then you need the experience to know how to apply it and when to apply it.” (W207)*

Another medical student also spoke of consultants with a *“repertoire of patterns”(W209)*:

, *E*xperience and ‘time served’ were not enough to guarantee wise decision-making, and certain other virtues were seen as key to phronetic decisions; particularly being reflective, *“open to insight” (WX04)* and being consultative, ‘”*it is always questioning what the right thing to do is and … get everybody’s views as well as their own. And respect everybody’s views. And be approachable as well… people being able to say, ‘Are you sure about that, boss, or shall we maybe try this?’” (B110).*

*Phronesis* was variously described as the collation of holistic information, both clinical and social, from different sources; and being able to weigh that up against protocols, guidelines, various situations encountered in the past and then getting other*“opinions, other approval, putting the situation to a new pair of eyes, and saying okay this is what I have got here.” (B106)*.

Many found *phronesis* hard to pin down, to define: wise decision-making and the skills employed to reach a wise decision were associated with a sort of gut instinct, what one GP called *“a sixth sense” (NX02),*

There are also occasions when making a wise decision means deferring the process. Some doctors spoke of the need to ‘buy time’ to reflect, to await more information, to take advice and thereby avoid making rash decisions, or prevent being unduly influenced by difficult conversations with the patient or family. It was about:

*“being deliberate, going slow;* *especially when dealing with patients and families from different cultures.” (WX06).*

An experienced doctor recounted a colleague who used a process named:

*“’MICLO’, which stands for ‘Masterly Inactivity with Cat-Like Observation’… because in some situations, inexperienced people would jump in to do things which weren’t necessarily the right thing to do, and that sometimes the wisdom is to actually sit back and watch what’s going on….” (NX01)*

Without resilience and time, and in the context of limited resources and information, less wise decisions are made. A foundation year doctor observed the difficulties of making an ethical decision around the withdrawal of active treatment, where more time and insight into the patient’s baseline and current functioning might have led to an earlier decision on withdrawal. The patient had been admitted at night and:

*“… it was much more difficult to get additional information from family members in the middle of the night and the team is also more pushed in terms of staffing at night… you don’t necessarily have the time to invest. The patient was transferred to another hospital for an operation but they didn’t operate for 24 hours, so actually, they did have the time that they could have done a bit of digging and then changed their mind; but I feel perhaps that element of, ‘We’ve already made the decision we’re going to operate, so we’re going to operate.’”. (N107, Follow-up)*

*Phronetic* decisions were seen as the avoidance of the rigid application of rules and guidelines, what was termed by one experienced doctor as the *“protocolisation of medicine” (WX03)*. A foundation year doctor described a consultant’s decision to not ‘red-card’ a drug user found injecting heroin in the hospital toilets, who was due a life-saving operation that day : *“the rules were applied but also there was some practical wisdom applied “ (B104). Similarly:“… it’s the human factors that we want from the doctors, and that’s why we can’t just use an app on your phone to work out your symptoms.” (N102)*

*Phronetic* decisions were therefore seen as practical and sensible approaches:

*“… you can learn everything about medicine, but if you don’t ever practice it you won’t develop the confidence or reinforce that knowledge in a way that is practical.” (WFY201)*

*“… where wisdom comes from, it’s a lot of thinking back to your past experiences and what you did badly, what you did well and trying to apply that… You’ve just got to think about in an ideal world what you want to do, and then think how you could possibly get as close to that with what you’ve got.” (B110 FP)*

# Reference
