# Supplementary material for: Using practical wisdom to facilitate ethical decision-making: a major empirical study of phronesis in the decision narratives of doctors
Source: BMC Med Ethics. 2021 Feb 18;22:16. doi: 10.1186/s12910-021-00581-y (PMC7890840; doi:10.1186/s12910-021-00581-y)
Supplement: Supplementary file 2 — Additional file 2: Title-Virtues from observation data. Description- the file shows the virtues observed during observation of MTD, in tabulated form. [file 12910_2021_581_MOESM2_ESM.docx]

# Virtues seen in observation data(Conroy, Hale et al. 2018)

| Virtue | Obs.1 | Obs.2 | Obs.3 | Obs.4 |
| --- | --- | --- | --- | --- |
| 1 | The way they do that is to explain all the options and the pros of cons of each one as they see autonomy as important and as recommended by NICE guidance. |  |  | “Dr W was clear and direct in explaining to the patient he would not be going home today. Dr W went out of the resus room and fetched the patient some water , he gave him two very small sips on separate occasions while he was with the patient and explained why he could not have more”. |
| 2 |  |  |  | “At the same time the consultant was aware of the priority patients allocated to the “top tray” and of other patients awaiting results in order to progress their care plans”.  Therefore judgement calls had to be made: to treat patients according to the severity of the disease. |
| 4 |  |  |  | “consultant .. on the urgent cases in the top tray and on the large computer screen on the wall which showed the bed state: also monitor the number of patients close to breaching national targets … they knew if would prempt issues later and problems for the patient tracker staff, but it was not always a priority.” |
| 5 | When familiar issues arise and they know what to do but when not familiar they seek advice in the form of guidance | An MDT to discuss 3 patients where all Registrar, Nurse physiotherapist and the OT gave their input to the consultant and then decided.  “there were concerns about patient and staff safety, restricting a patient’s liberty, capacity vs incapacity, concerns about understanding information”,  “ a lot of searching for consensus” | “There is a lot of discussion between the doctors and almost nothing from the nurses or the therapists ….” | “Where a lot of rapid decisions are being made and where humour, mutual support and compassion were on display”. |
| 7 |  | “members of the MDT had an increased level of caring for certain patients based on the levels of concern they exhibited, the length of the discussion, their facial expressions and how they referred to them”…  “There was no animosity or anger towards difficult patients, even when they were abusive or harassing”…. |  | “compassion [for patients]were on display”.  “Dr W introduced himself to the patient , spoke to the doctor and then came close to the patient so he could hear him and spoke .. what may be happening and what would happen next. This seemed to enable the patient to speak about his concerns which were when he could go home and the fact that his mouth was very dry.  Dr W was clear and direct in explaining to the patient he would not be going home today. Dr W went out of the resus room and fetched the patient some water , he gave him two very small sips on separate occasions while he was with the patient and explained why he could not have more. |
| 9 |  |  |  | “Dr W stood or walked the department for the observation apart from a short discussion when he sat down with a doctor at her suggestion to discuss an older patient with complex health and social problems”. |
| 10 |  | Medical and social goal oriented discussions |  | “discussion when he sat down with a doctor at her suggestion to discuss an older patient with complex health and social problems. “ |
| 11 |  |  | “Initially a start in the summary from the registrar, then one of the doctors would interrupt if they had a question. There was a lot of story sharing from the doctors, a sort of working out their internal thought processes out loud”. |  |
| 15 |  |  | “This baby didn’t have the classic signs of a particular disease, necrotizing enterocolitis (NEC), but it turned out she had it, and they only found it because the doctors persisted on checking things because her symptoms were odd but consistent.”… | Judgement call should to be made to treat patients according to the severity of illness and for this experience/ practical wisdom is required |
| 14 |  | “quite a lot of concerns about resource allocation and restrictions” |  | A scanner was loaned out to another Dept. Arrangements were made to support paediatric A&E although the two A&E consultants discussed the need to not let this become a pattern … |
| Manage a busy schedule |  |  | + |  |
| Seeking reassurance |  | + | + |  |
| Earnest in solving problems |  | + | + |  |
| Get the details of each case | + |  |  |  |
| Guidelines are important | + | + | + |  |
| Leadership |  |  |  | + |

# Reference

Conroy, M., C. Hale, A. Malik, C. Weir, D. Biggerstaff, A. Brockie, C. Turner and R. Knox. (2018). "Phronesis in Medical Decision Making: Medical Leadership, Virtue Ethics and Practical Wisdom. AHRC Final Report for Phronesis and the Medical Community." 2019, from <https://www.birmingham.ac.uk/Documents/college-social-sciences/social-policy/phronesis/phronesis-in-medical-decision-making.pdf>.
